# Histamine Intolerance: An Overlooked Diagnosis of Recurrent Anaphylaxis-Like Symptoms

**DOI:** 10.7759/cureus.80649

**Published:** 2025-03-16

**Authors:** Takashi Nakamura, Hirohisa Fujikawa, Hirotake Ikeda, Satoshi Mizuma

**Affiliations:** 1 Department of Internal Medicine, Suwa Central Hospital, Nagano, JPN; 2 Center for General Medicine Education, School of Medicine, Keio University, Tokyo, JPN

**Keywords:** allergy and anaphylaxis, fish allergy, food-induced anaphylaxis, food intolerance, histamine fish poisoning, histamine intolerance, histamine receptors, scombroid fish poisoning

## Abstract

Histamine intolerance is a common disorder associated with an impaired histamine metabolism. It should be considered in individuals with sporadic or repeated anaphylaxis-like symptoms after seafood consumption. We encountered a 26-year-old male patient who experienced recurrent episodes of anaphylaxis-like symptoms after ingesting seafood. A detailed history-taking and a histamine 50-skin-prick test confirmed the diagnosis of histamine intolerance. In addition, a low-histamine diet and prophylactic antihistamine medication did not result in symptom recurrence. Proper management of histamine intolerance can improve the individual's quality of life.

## Introduction

Histamine intolerance is a condition in which various symptoms related to histamine receptors occur due to the accumulation of histamine in the body, which results from a decreased ability to break down histamine [1,2]. Anaphylaxis and histamine poisoning are differential diagnoses, but histamine intolerance should be considered in individuals with sporadic or repeated anaphylaxis-like symptoms after ingesting seafood. Histamine intolerance is diagnosed comprehensively based on medical history, diagnostic therapeutic interventions, and additional tests, with the histamine 50-skin-prick test being particularly useful [3,4]. Management includes a low-histamine diet and antihistamines, which can improve the individual's quality of life [1,2]. A low-histamine diet should be continued for at least one month until symptoms are resolved [3,5]. Diamine oxidase (DAO) supplements are being studied as a complementary therapy for histamine intolerance and are available without a prescription in some countries [1].

Here, we report a case of histamine intolerance presenting with recurrent anaphylaxis-like symptoms after seafood ingestion. The findings of this report will increase awareness of this condition among clinicians.

## Case presentation

A 26-year-old man with no known medical history or allergies presented to the emergency department with a one-hour history of generalized pruritic rash, pharyngeal discomfort, dyspnea, headache, and diarrhea. There were no findings suggestive of infectious gastroenteritis, such as fever, abdominal pain, or vomiting. The symptoms started two hours after a dinner which consisted of tuna, salmon, whelk shellfish, and octopus at a sushi restaurant. None of the individuals around him experienced similar symptoms.

On examination, the patient's temporal temperature was 36.6°C, heart rate was 77 beats per minute, blood pressure was 112/86 mm Hg, respiratory rate was 22 breaths per minute, and oxygen saturation was 96% while breathing ambient air. Physical examination revealed generalized erythema, mainly on the trunk; however, he did not have other signs. Seafood-induced anaphylaxis was suspected, and the patient was treated with intramuscular epinephrine (0.3 mg), intravenous chlorpheniramine (5 mg), famotidine (20 mg), and methylprednisolone (80 mg). The patient's symptoms improved rapidly after treatment.

**Figure 1:**
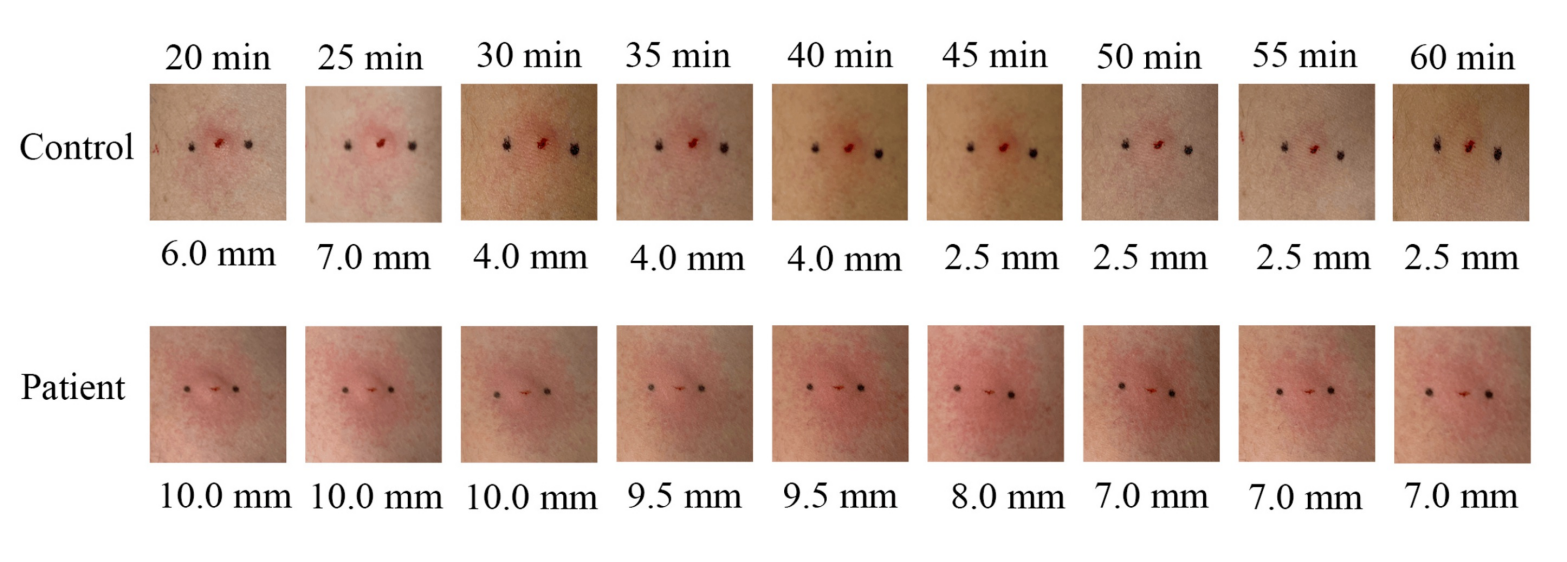
Changes in the size of wheals in the histamine 50-skin-prick test.

However, over the next four years, he had four similar presentations to the emergency department and was treated for anaphylaxis (Table 1). We performed a serum test on the patient to rule out allergic diseases and anaphylaxis, which showed negative for immunoglobulin E (IgE) antibodies to seafood and anisakis. Although histamine poisoning was considered a differential diagnosis, the symptoms were atypical in that they were recurrent and did not occur in clusters. Although serum tryptase levels were not measured, all symptoms were clearly triggered by seafood consumption, and the possibility of mast cell activation syndrome was considered unlikely. Accordingly, we suspected histamine intolerance, and the patient underwent a histamine 50-skin-prick test. The size of the wheal at 50 minutes was greater than 3 mm (Figure 1), supporting the diagnosis of histamine intolerance. Since then, he has been on a low-histamine diet and prescribed prophylactic oral antihistamines at the time of seafood intake. Notably, the symptoms have not recurred for approximately four years to date.

**Table 1:** Orally ingested foods, time to onset, and symptoms associated with histamine intolerance.

Food	Time to onset	Symptoms
Tuna, salmon, whelk, octopus	2 hours	Flush, pruritus, oral discomfort, dyspnea, diarrhea
Tuna, whelk	4 hours	Flush, pruritus, oral discomfort, abdominal pain, diarrhea
Yellowtail, cod	30 minutes	Flush, wheal, pruritus, dyspnea, tachycardia, diarrhea
Salmon, greater amberjack, sea bream	1 hour	Flush, pruritus, fatigue, headache

## Discussion

In this case, a patient who presented with anaphylaxis-like symptoms after ingesting seafood underwent thorough history-taking and the histamine 50-skin-prick test, which provided an objective assessment of the reduction in histamine reactivity and led to the diagnosis of histamine intolerance. In addition, a low-histamine diet and prophylactic antihistamine medication did not result in symptom recurrence. Thus, the present case illustrates the importance of an appropriate diagnosis of histamine intolerance, which can contribute to improving a patient's quality of life.

Histamine intolerance is a relatively common but unrecognized disorder associated with an impaired histamine metabolism. It has a cumulative prevalence of 1-6% and frequently manifests during childhood or middle age [1-3]. It is defined as a disorder that results from a reduction in the histamine degradation capacity of the intestine due to either congenital or acquired impaired DAO activity [3]. Acquired causes include gastrointestinal disorders, such as inflammatory bowel disease and functional disorders, and medications such as verapamil and isoniazid [5]. This leads to the accumulation of histamine in the plasma and the subsequent manifestation of symptoms [1,2]. Food intolerance is caused by the accumulation and ingestion of minute amounts of histamine and is not a problem in healthy individuals [3]. Therefore, the condition differs from histamine poisoning, which occurs after the ingestion of foods that contain high levels of histamine. It also differs from anaphylaxis, which is defined as an excessive endogenous release of histamine and other chemicals via immunological mechanisms due to exposure to triggers or direct stimulation of mast cells and basophils [1,6]. Mast cell activation syndrome also presents with a variety of similar symptoms, but histamine intolerance differs in that histamine action is excessive without excessive mast cell activation [7].

The symptoms and manifestations of histamine intolerance are diverse and nonspecific. The symptoms occur according to the histamine receptors and affect the dermatological, respiratory, gastrointestinal, cardiovascular, and nervous systems [3]. The most common gastrointestinal symptoms are bloating (92%), diarrhea (71%), and abdominal pain (68%), and the associated skin symptoms include pruritus, erythema, flushing, and swelling [8]. Intolerance also causes respiratory symptoms such as dyspnea, nasal congestion, rhinorrhea, and sneezing and affects the nervous system, with headaches (65%) presenting more commonly than allergies [8,9]. Thus, the diagnosis of histamine intolerance presents a significant challenge for clinicians because of the extensive range of clinical manifestations. However, it is precisely this variety, unpredictability, and randomness of the symptoms that strongly suggest the presence of histamine intolerance and provide a clue for further investigation.

Due to the absence of a consensus on diagnostic criteria, histamine intolerance is comprehensively diagnosed based on history, diagnostic therapeutic interventions, and additional tests (Table 2) [3]. Of the additional tests for the diagnosis of histamine intolerance, the histamine 50-skin-prick test is the one that is safe and can be performed under the Japanese health insurance system. This cannot distinguish between histamine intolerance and other allergic diseases. It should therefore only be used for making a definitive diagnosis and has recently become widely used [2,4,5,10,11]. The test is performed as follows: "A 1% (10 mg/mL) histamine solution is pipetted onto intact skin and the area is pricked with lancets. If a wheal >3 mm is present at 50 minutes after puncture, the test result is positive" [4]. The delay in the disappearance of the wheal is associated with a decrease in DAO activity and suggests a decrease in histamine degradation capacity, and this has recently been used across the world [4,12]. Due to the similarity in the presentation of dermatological, respiratory, cardiovascular, and gastrointestinal symptoms after seafood ingestion, it is clinically important to differentiate histamine intolerance from anaphylaxis, histamine poisoning, and mast cell activation syndrome. In contrast to anaphylaxis, specific IgE test results within the normal range and headaches are characteristic findings in patients with histamine intolerance [1,9]. In contrast to histamine poisoning, histamine intolerance is characterized by sporadic or recurrent onset, along with a relatively long time to onset after seafood ingestion (less than four hours) [2,13]. In contrast to mast cell activation syndrome, histamine intolerance is usually limited to postprandial symptoms, especially after seafood consumption, with normal serum tryptase levels after the onset of symptoms [14].

**Table 2:** Approaches for the definitive diagnosis of histamine intolerance. DAO: diamine oxidase; HNMT: histamine N-methyltransferase

Approaches to definitively diagnose histamine intolerance	
History	Presenting ≥2 symptoms of histamine intolerance
	Manifestation of symptoms in less than four hours after food intake
	Exclude other diseases
Diagnostic therapy (4-8 weeks)	Symptoms improved after dismissing drugs interfering with histamine metabolism and distribution
	Symptoms improved after using H1/H2 antihistamines
	Symptoms improved after low-histamine diet
	Symptoms improved after DAO supplementation
Additional tests	Histamine 50-skin-prick test
	Oral histamine-challenge test
	Determination of histamine and its metabolites in urine or stool
	Determination of histamine in blood
	Single-nucleotide polymorphism of DAO/HNMT gene assessment

A multifaceted approach is required to treat histamine intolerance, and a low-histamine diet is the gold standard for the management of histamine intolerance [1]. A low-histamine diet should be continued for at least one to two months until symptoms are resolved [3,5]. Histamine-rich foods include aged cheese, avocado, chocolate, fermented foods, and seafood, while low-histamine diets include bread, eggs, rice, fresh juice, herbal tea, and water [1]. Additionally, foods that promote the release of histamine, such as citrus fruits, pork, cheese, chocolate, and fish, and foods that competitively inhibit DAO, such as fermented sausage, sauerkraut, and fish, should be avoided. Seafood is rich in histamine, promotes the release of histamine, and inhibits DAO [1]. DAO supplementation is also considered useful for treating affected individuals [2]. Despite a lack of direct evidence, it is reasonable to consider the prophylactic use of second- or third-generation H1 antihistamines (e.g., fexofenadine hydrochloride, loratadine, desloratadine, bilastine) in situations where histamine ingestion is unavoidable [1].

## Conclusions

Herein, we report a case of histamine intolerance with recurrent anaphylaxis-like symptoms after ingesting seafood. There are likely many such cases in which anaphylaxis-like symptoms of unknown cause have been repeatedly documented but not correctly diagnosed. Histamine poisoning is an important differential diagnosis for idiopathic anaphylaxis after seafood ingestion. However, in the present patient, histamine intolerance was considered because of the lack of outbreaks and symptom recurrence.

Histamine intolerance is relatively common, but it is thought that there are still few cases that have been properly diagnosed. Although there are no established diagnostic criteria and guidelines, most cases are diagnosed and treated using the management approaches described in the review literature, as in this patient. The appropriate diagnosis and therapeutic intervention for histamine intolerance will contribute to improving the quality of life of affected individuals around the world.
